# NADPH-Independent Fluorescent Probe for Live-Cell Imaging of Heme Oxygenase-1

**DOI:** 10.1021/acssensors.4c02978

**Published:** 2025-01-02

**Authors:** Liang Li, Xuanyi Lu, Qiyuan He, Chao Shu, Edward R. H. Walter, Lin Wang, Nicholas J. Long, Lijun Jiang

**Affiliations:** † Hubei Key Laboratory of Genetic Regulation & Integrative Biology, Key Laboratory of Pesticide and Chemical Biology of Ministry of Education, School of Life Sciences, Central China Normal University, Wuhan 430079, China; ‡ State Key Laboratory of Green Pesticide, College of Chemistry, Central China Normal University, Wuhan 430079, China; § Department of Chemistry, Imperial College London, MSRH Building, White City Campus, London W12 0BZ, U.K.; ∥ Institute of Systems Medicine, Chinese Academy of Medical Sciences, Suzhou 215028, China

**Keywords:** molecular probe, fluorescence, heme oxygenase-1, peptides, protein imaging

## Abstract

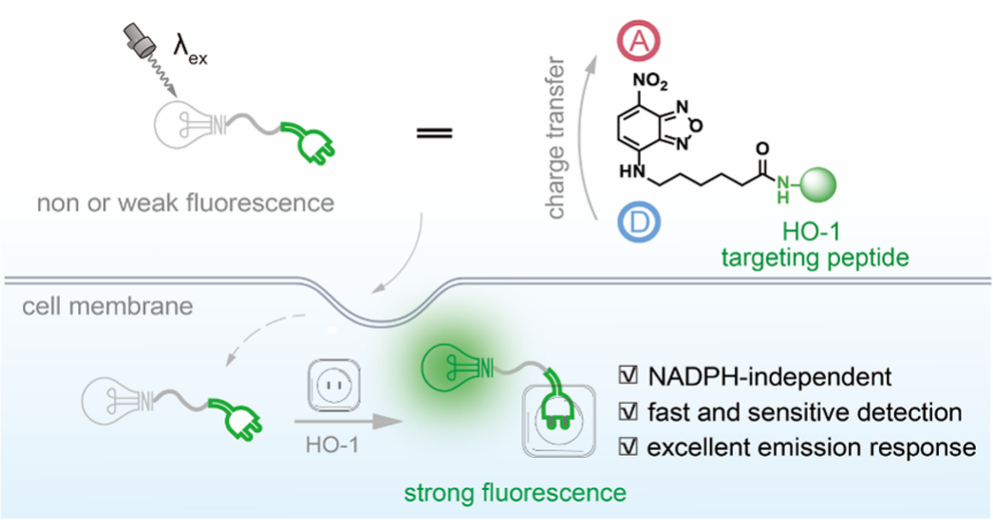

Heme oxygenase-1 (HO-1) catalyzes heme degradation on the consumption of NADPH and molecular oxygen. As an inducible enzyme, HO-1 is highly induced in various disease states, including cancer. Currently, two fluorescent probes for HO-1 have been designed based on the catalytic activity of HO-1, in which the probes serve as a substrate, so NADPH is required to enable the detection. Probes functioning in a NADPH-dependent way may influence other NADPH-consuming pathways, as all these pathways share a common NADPH pool. Here, we report the peptide-based fluorescent probe NBD-P_5_ as a simple alternative approach for HO-1 sensing. The designed probe NBD-P_5_ functions independently of the catalytic activity of HO-1, therefore enabling fast and sensitive detection of HO-1 with no requirements of other substances, including NADPH and biliverdin reductase. Moreover, it overcomes the need for a large substrate amount and long incubation time during the detection. NBD-P_5_ can be quickly taken up by cells, demonstrates an excellent colocalization with the endoplasmic reticulum (where HO-1 is mainly located), and is shown to be reliable in reporting changes in HO-1 levels in live cells. This work provides a simple alternative approach for designing HO-1 fluorescent probes, and we expect it will act as a practical tool for further studying HO-1 biology.

Heme oxygenase-1 (HO-1), an inducible enzyme, catalyzes the degradation of free heme (Fe(III) protoporphyrin IX) using reducing equivalents from NADPH-dependent cytochrome P450 reductase.^1^ Free heme, also known as labile heme, refers to a heme that is not or is weakly bound to proteins. Free heme is toxic due to its ability to provide redox-active iron that can promote oxidative stress. Catalytic action toward the end products including biliverdin, Fe(II), and carbon monoxide, as well clearance of the cytotoxic heme, imparts HO-1 antioxidant and anti-inflammatory effects.^2^ Although the basal HO-1 levels in healthy tissues are low, as an inducible protein, HO-1 is highly induced in cancer cells. The overexpression is commonly seen in a wide range of cancer types to favor their survival, proliferation, invasion, metastasis, resistance to anticancer therapy, and modulating tumor microenvironment.^3^ Notably, HO-1 expression level is positively related to the disease stage and poor prognosis.^4^ Therefore, it holds the potential to serve as a biomarker for not only cancer diagnosis but also cancer prognosis and treatment monitoring.^5−7^ Despite the biological importance of HO-1 in heme breakdown and cancer progression, many of the exact mechanisms of action involved remain unclear, for example, the specific mechanism of HO-1 translocation into the nucleus.^8^ The lack of progress in research on HO-1 biology is primarily due to a lack of tools and methods for HO-1 monitoring. Traditional methods toward HO-1 detection are mainly performed by measuring the degradation products following catalysis by HO-1 via spectroscopy,^9^ high-performance liquid chromatography (HPLC),^10,11^ or radiochemistry,^12^ which is cumbersome to operate and cannot be applied for live-cell HO-1 imaging in principle. Monitoring of cellular HO-1 is crucial to understanding its dynamics and functions in a biological system, which largely depends on the availability of suitable fluorescent probes.

Very recently, two fluorescent probes Fe-L^1^ and RBH were designed for HO-1 activity.^13,14^ Both probes are based on the catalytic action of HO-1, which blocked the preexisting energy transfer via either the fluorescence resonance energy transfer (FRET) mechanism or the static quenching mechanism (Scheme 1A,B). Furthermore, RBH is able to image live-cell HO-1. However, the reliability and sensitivity of such enzyme-activable fluorescent probes are affected by enzyme activity as well as the concentration of NADPH and O_2_. For example, the low oxygen level in the tumor microenvironment presents a barrier for accurate HO-1 sensing, and their activity may influence other NADPH- and O_2_-consuming pathways, as all these pathways use a common NADPH pool. Finally, a large probe concentration (40–50 μM) along with a long incubation time (4–16 h) was used to enable a fluorescence response of each of the two probes. It is, therefore, highly beneficial to design new fluorescent probes responding to HO-1 quickly and sensitively in live cells to improve cancer diagnosis.

**Scheme 1:**
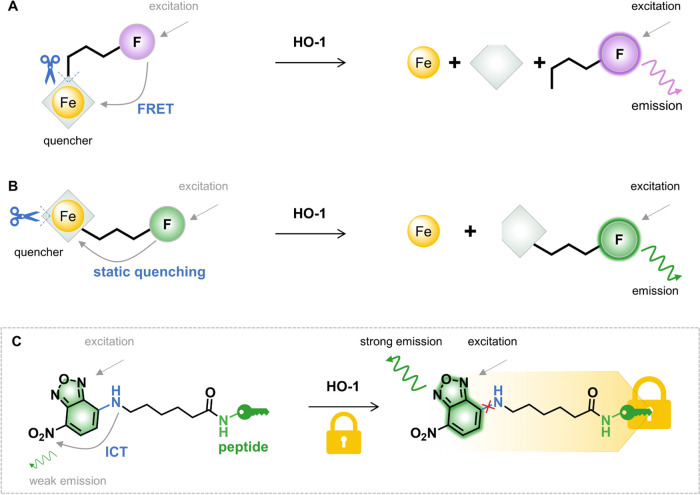
Schematic Illustration of the Sensing Mechanism of the HO-1-Responsive Fluorescent Probe (A) Fe-L^1^,^13^ (B) RBH,^14^ and (C) NBD-P_5_

Peptides can serve as an ideal recognition element for many types of cancer, as they offer high natural specificity toward the target by mimicking endogenous substrate, so a peptide-based fluorescent probe is an alternative approach for the detection of protein biomarkers.^15,16^ In our continuous efforts to employ peptides to construct probes for imaging and regulation of proteins,^17−20^ we have overcome the dependence on NADPH and O_2_ for HO-1 detection by reporting a peptide-based fluorescent probe NBD-P_5_ that acts independently of the catalytic ability of HO-1. Probe NBD-P_5_ enabled the sensitive and fast-responsive detection of HO-1 and maintained the fluorescent responsiveness (9-fold fluorescence increase). Also, it is capable of reporting changes in HO-1 levels in live cells. We expect that NBD-P_5_ will be a practical chemical tool for gaining a better understanding of HO-1 biology.

## Results and Discussion

### Design, Synthesis, and Characterization of NBD-P*_n_*

HO-1 is a typical caveolin-1 binding protein. Caveolin-1 is a major structural protein of caveolae, and colocalization of caveolin-1 with HO-1 in caveolae has long been observed.^21^ The minimum binding sequence of caveolin-1 to HO-1 was shown to be a pentapeptide Y_97_WFYR_101_.^22^ A subsequent study showed that deletion of a 101 arginine residue (Y_97_WFY_100_) can also bind to the HO-1 protein.^23^ The two peptides are then employed and modified to construct HO-1 binding fluorescent probes. To achieve a fluorescence response toward HO-1 binding, a nitrobenzoxadiazole (NBD) unit was chosen as a model fluorophore to study interactions between HO-1 and the probes due to its well-studied intramolecular charge transfer (ICT) characteristics.^24,25^ The ICT-characterized fluorophores have high photosensitivity toward microenvironmental perturbations, including binding by proteins; therefore, NBD-based fluorescent probes have been frequently exploited for sensing proteins.^26,27^ Therefore, two HO-1 responsive fluorescent probes, NBD-P_4_ and NBD-P_5_, were designed (P_4_: YWFY-CONH_2_, P_5_: YWFYR-CONH_2_; Scheme 1C).

**Figure 1:**
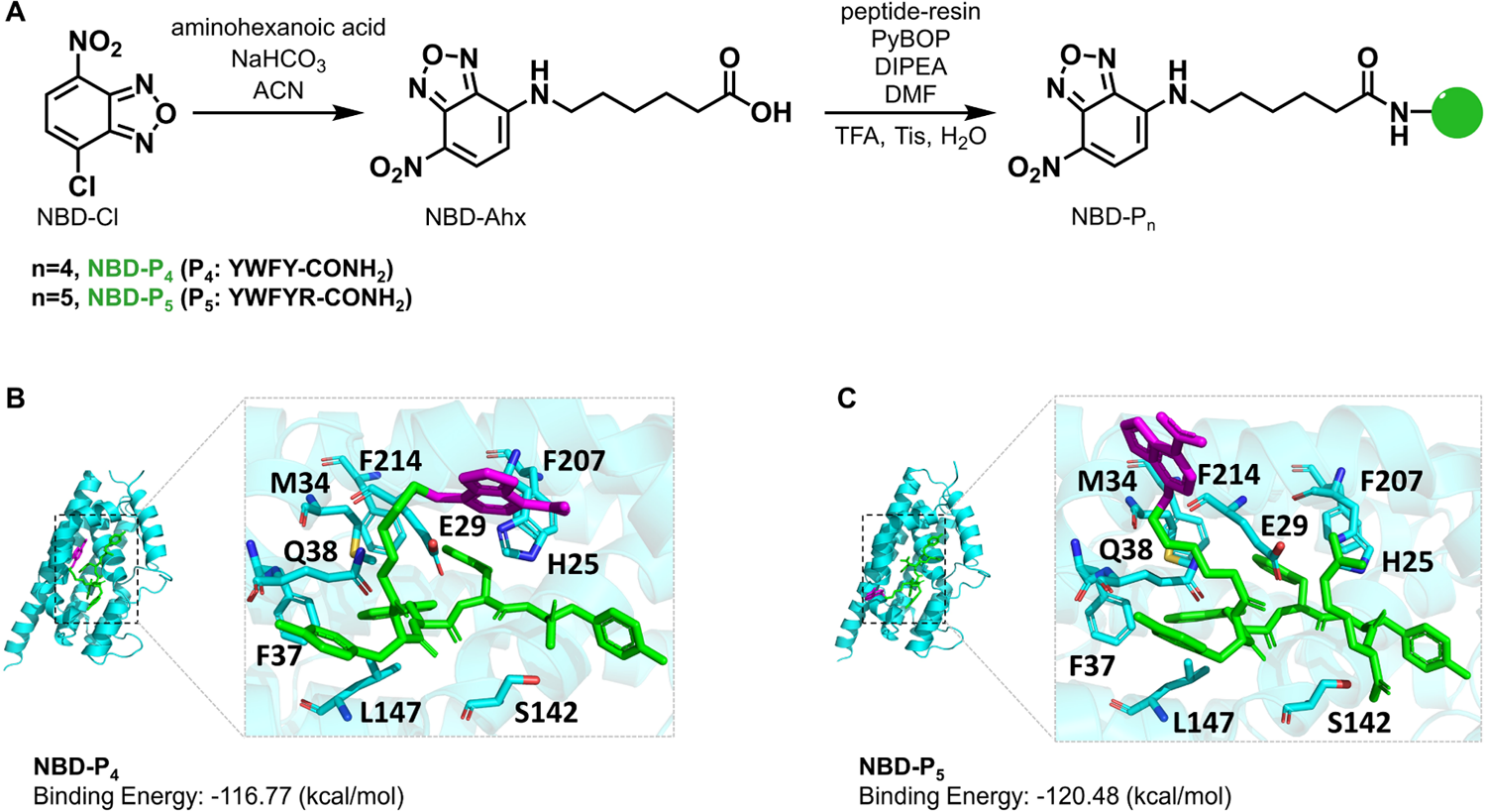
Synthetic route toward NBD-P*_n_* (*n* = 4, 5) and their binding modes with HO-1. (A) The two-step synthetic route of NBD-P*_n_*. (B, C) The predicted binding poses and binding energies between HO-1 and NBD-P*_n_* were obtained by computational modeling. The fluorophore moiety is shown in purple with the peptide moiety in green. Key residues in HO-1 forming strong interactions with NBD-P*_n_* are labeled and shown in stick.

A simple two-step synthetic route was conducted to synthesize NBD-P*_n_* (Figure 1A). The first step to produce NBD-Ahx (Ahx: 6-aminohexanoic acid) was carried out according to a previous report.^28^ Ahx was used as a linker to provide flexibility to the resulting probes and reduce the impact of the fluorophore conjugation on interactions between the protein and the peptides. NBD-Ahx was then coupled to P_4_ and P_5_ using PyBOP under basic conditions. The obtained peptide conjugates were subjected to trifluoroacetyl (TFA) cleavage and purified using reversed-phase HPLC. Characterization of intermediates and probes was performed, as shown in Figures S1–S7.

### Computational Studies of the Interaction between HO-1 and NBD-P*_n_*

To see if NBD-P_4_ and NBD-P_5_ can bind to HO-1, in silico modeling studies were performed between the two probes and the HO-1 protein using AlphaFold3^29^ and docking simulations. The structural features of HO-1 are summarized in Figure S8. As shown in Figure 1B,C, although the ligand conformation differs from each other, especially the fluorophore moiety, both share similar interactions with key HO-1 residues, including H25, E29, M34, F37, Q38, S142, L147, F207, and F214. The two side chains of amino acids W and F in the probe ligand form hydrophobic interactions with M34, F37, L147, F207, and F214 of HO-1. H25, E29, Q38, and S142 in the HO-1 protein form a hydrogen bond network with the amide bonds in the probe ligand, further improving the stability of the complex structure. Both ligands were suggested to interact hydrophobically with the two key residues, F207 and F214, of the heme-binding pocket. This observation is consistent with findings by Higashimoto and co-workers, where they showed that F207 and F214 were in the motif bound by the peptide deriving from caveolin-1.^22^ Additionally, the arginine in P_5_ forms additional hydrogen bonds with E29 of HO-1.

By molecular mechanics and general Born surface area (MM/GBSA) calculations, both probes were predicted to potentially be ligands for HO-1 with low binding energy (high binding affinity). NBD-P_5_ demonstrates a slightly stronger affinity due to the additional contribution of arginine. Similarly, P_5_ is suggested to have a stronger binding to HO-1 than to P_4_. As expected, the conjugated NBD fluorophore did not weaken the binding affinity of the peptides to HO-1 (Figure S9).

### Photophysical Properties of NBD-P*_n_*

**Figure 2:**
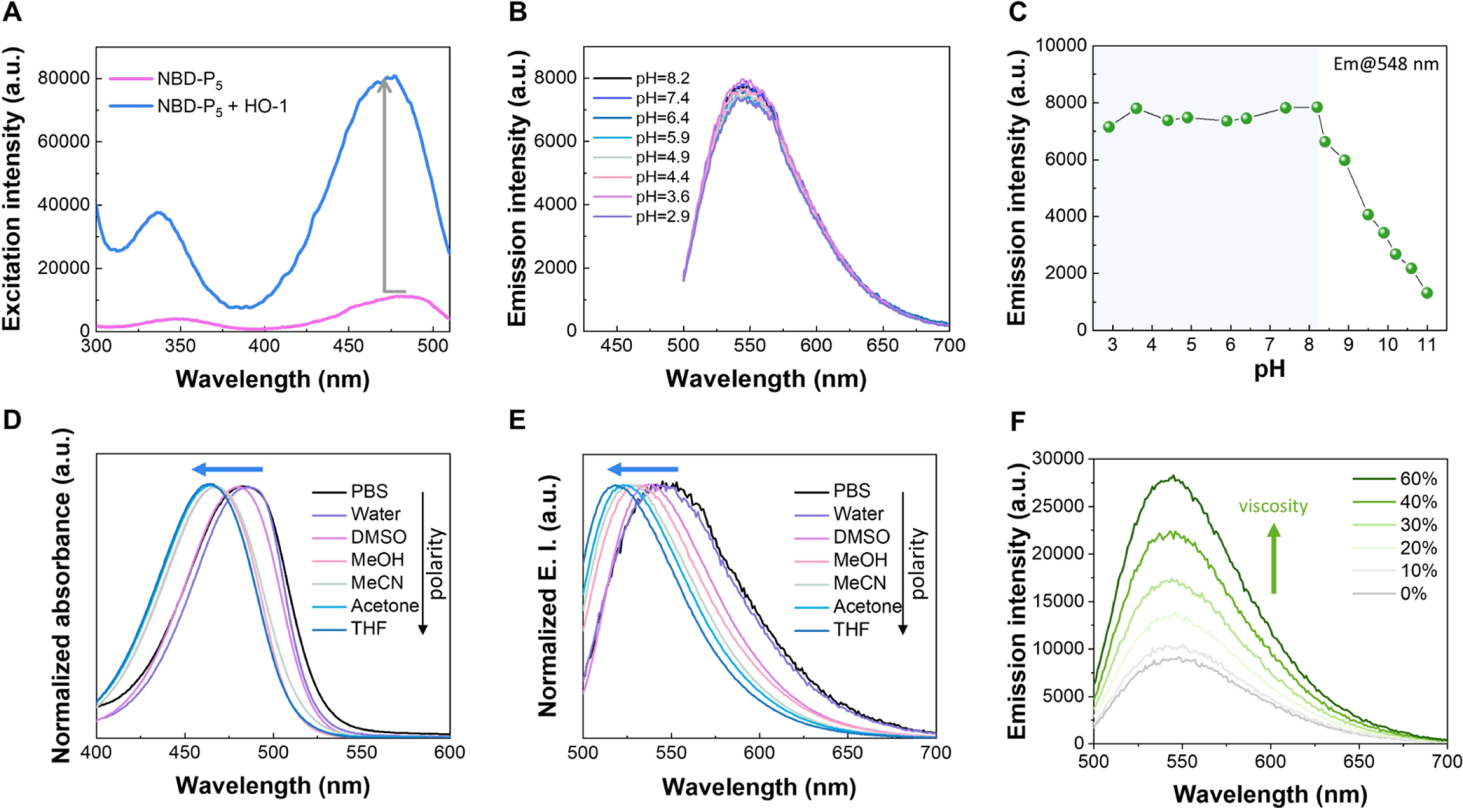
(A) Excitation profile of NBD-P_5_ recorded at λ_em_ = 535 nm before and after the addition of HO-1. (B) Emission profile of NBD-P_5_ and (C) emission intensity of NBD-P_5_ at 548 nm under different pH conditions. (D) Normalized absorption and (E) emission spectra of NBD-P_5_ in solvents of different polarities. (F) Emission profile of NBD-P_5_ in different volume ratios of water and glycerol. λ_ex_ = 475 nm, [NBD-P_5_] = 2.0 μM, [HO-1] = 4.5 μM.

We next examined the binding of NBD-P*_n_* toward HO-1 in solution and whether it can facilitate a spectral change. First, the excitation of NBD-P*_n_* before and after the addition of HO-1 was measured and compared. The recombinant HO-1 protein with a 6xHis tag was used in this study. As shown in Figure 2A, an enhanced and blue-shifted excitation spectrum was presented with HO-1, suggesting that the binding action to HO-1 inhibits the ICT process of NBD-P_5_. A similar but weaker trend in changes of excitation was observed for NBD-P_4_ (Figure S10), and this is consistent with the docking results. The ICT characteristics of NBD-P*_n_* were, therefore, evaluated through a spectroscopic study using solvents with varying polarities. The gradual blue shifts in both absorption and emission maxima were displayed upon decreasing solvent polarity from phosphate-buffered saline (PBS)/water to tetrahydrofuran (THF; Figures 2D,E and S11). Similarly, solvent effects on the spectral profile of NBD-P_4_ were studied, as shown in Figures S12 and S13. We also evaluated the emission profile of NBD-P_5_ in solvents with varying viscosities. As demonstrated, the fluorescence gradually increased with the increase in viscosity (Figure 2F). These experiments confirm the strong ICT character of NBD-P*_n_*.

The pH sensitivity of NBD-P*_n_* was checked before examining the fluorescence responsiveness. Although HO-1 is primarily located in the endoplasmic reticulum (neutral, pH 7.2), it has also been shown to localize at other cellular compartments, including caveolae, nucleus, and mitochondria (where the pH is found to be quite alkaline to ∼8.0).^30,31^ Therefore, it is important that the fluorescence signal of the probe is not influenced by the pH range from neutral to mildly alkaline. As illustrated in Figure 2B,C, the emission profile of NBD-P_5_ at different pH conditions was measured, which showed almost no change toward pH variations from 2.9 to 8.2 (NBD-P_4_ kept stable at pH 2.2–8.3, Figures S14 and S15). The emission decrease of NBD derivatives in an alkaline environment has been previously described.^32^ This can be attributed to the increase in OH^–^ ions, which restricts the intramolecular charge transfer within the fluorophore, thereby weakening the ICT excited state and its emission intensity. The result indicates that NBD-P*_n_* is pH-insensitive over physiological pH ranges.

### NBD-P_5_ Responds to HO-1 Highly and Selectively

**Figure 3:**
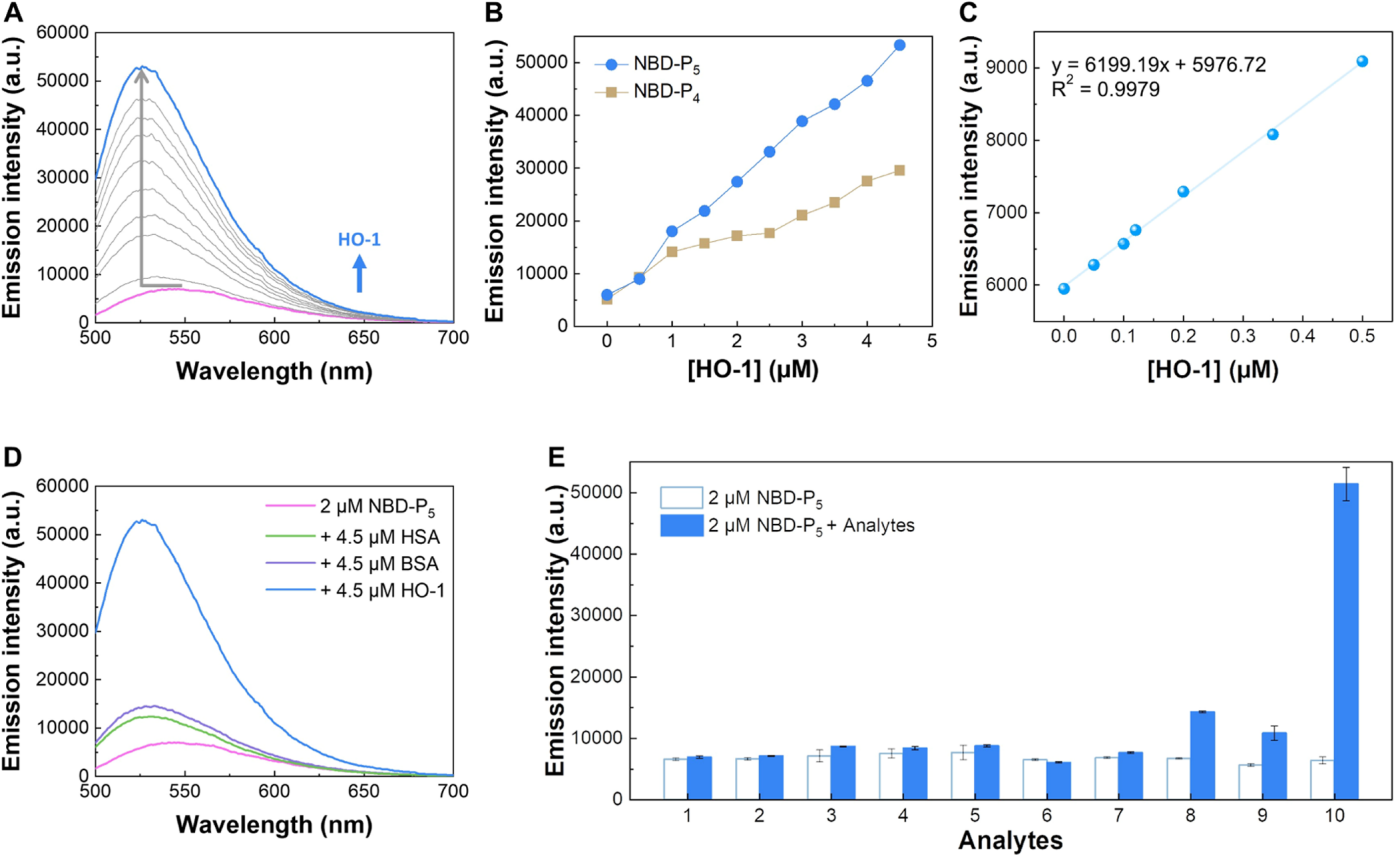
NBD-P_5_ responds to HO-1 with high selectivity. (A) Emission profile of NBD-P_5_ in responding to HO-1 with increasing concentrations. (B) Changes in the emission intensity of NBD-P_4_ and NBD-P_5_ at 526 nm on addition of HO-1. (C) The linear relationship between the emission intensity of NBD-P_5_ at 526 nm and the HO-1 concentration is in the range of 0–0.5 μM. (D) Changes in the emission profile of NBD-P_5_ with HSA, BSA, and HO-1 under the same concentration of 4.5 μM. (E) Selectivity of NBD-P_5_ to HO-1 among biologically relevant ions, amino acids, and common proteins. Bars represent the fluorescence intensity of NBD-P_5_ at 526 nm before and after addition of analytes. The concentration is 1.0 mM for 1. NaCl, 2. KCl, 3. NaHCO_3_, 4. KH_2_PO_4_, 5. Ala, 6. Lys, and 7. GSH. The concentration is 4.5 μM for 8. BSA, 9. HSA, and 10. HO-1. λ_ex_ = 475 nm, [NBD-P_4_] = 2 μM, [NBD-P_5_] = 2 μM. All measurements were taken within a minute after the addition of the analyte.

We then moved to check the reactivity and fluorescence response of NBD-P_5_ toward HO-1. NBD-P_5_ alone was weakly fluorescent. Similar to changes in the excitation spectrum, a gradually increased and blue-shifted emission was demonstrated upon HO-1 addition and in a concentration-dependent manner. This is attributed to the restriction of intramolecular motion of NBD-P_5_ by the bound HO-1. Addition of 4.5 μM HO-1 to NBD-P_5_ caused a 9-fold increase in fluorescence intensity and a 24 nm blue shift in fluorescence wavelength maxima (Figure 3A). NBD-P_4_ also responded to HO-1, but the fluorescence responsiveness was weaker compared to that of NBD-P_5_, accounting for a 6-fold increase in fluorescence intensity (Figures S16A and 3B). According to the linear relationship found for the emission intensity of NBD-P_5_ at 526 nm and the HO-1 concentration in the range of 0–0.5 μM (Figure 3C), a detection limit of NBD-P_5_ for HO-1 was calculated as 0.036 μM using the 3σ method. Note that every measurement was taken within 1 min after the addition of HO-1, and HO-1 was the only added substance for the fluorescence response. Thus, NBD-P_5_ can quickly respond to HO-1 with improved sensitivity and fluorescence responsiveness compared to the two previously reported probes that are based on the catalytic activity of HO-1 (6-fold increase with 50 μM probe after 16 h, or 4-fold increase with 40 μM probe after 4 h, and biliverdin reductase and NADPH were additional supplemented for both probes to function).

Selectivity toward the analyte is one of the most important features to consider for fluorescent imaging probes. Therefore, we investigated the selectivity of NBD-P*_n_* toward HO-1 against various potential interfering substances, including two common proteins, amino acids, and biologically relevant ions. One of the proteins is human serum albumin (HSA), the most abundant circulating protein in the blood plasma. As presented in Figure 3D,E, addition of HSA and bovine serum albumin (BSA) caused a small fluorescence increase, which can be attributed to the nonspecific binding, suggesting the binding of NBD-P_5_ for them was weaker than for HO-1. Virtually no substantial change in fluorescence was observed with intracellular ions and amino acids. Using analogous experiments, selectivity toward HO-1 by NBD-P_4_ was also investigated (Figure S16B,C).

### NBD-P_5_ Can Image Endoplasmic Reticulum HO-1 in Live Cells

**Figure 4:**
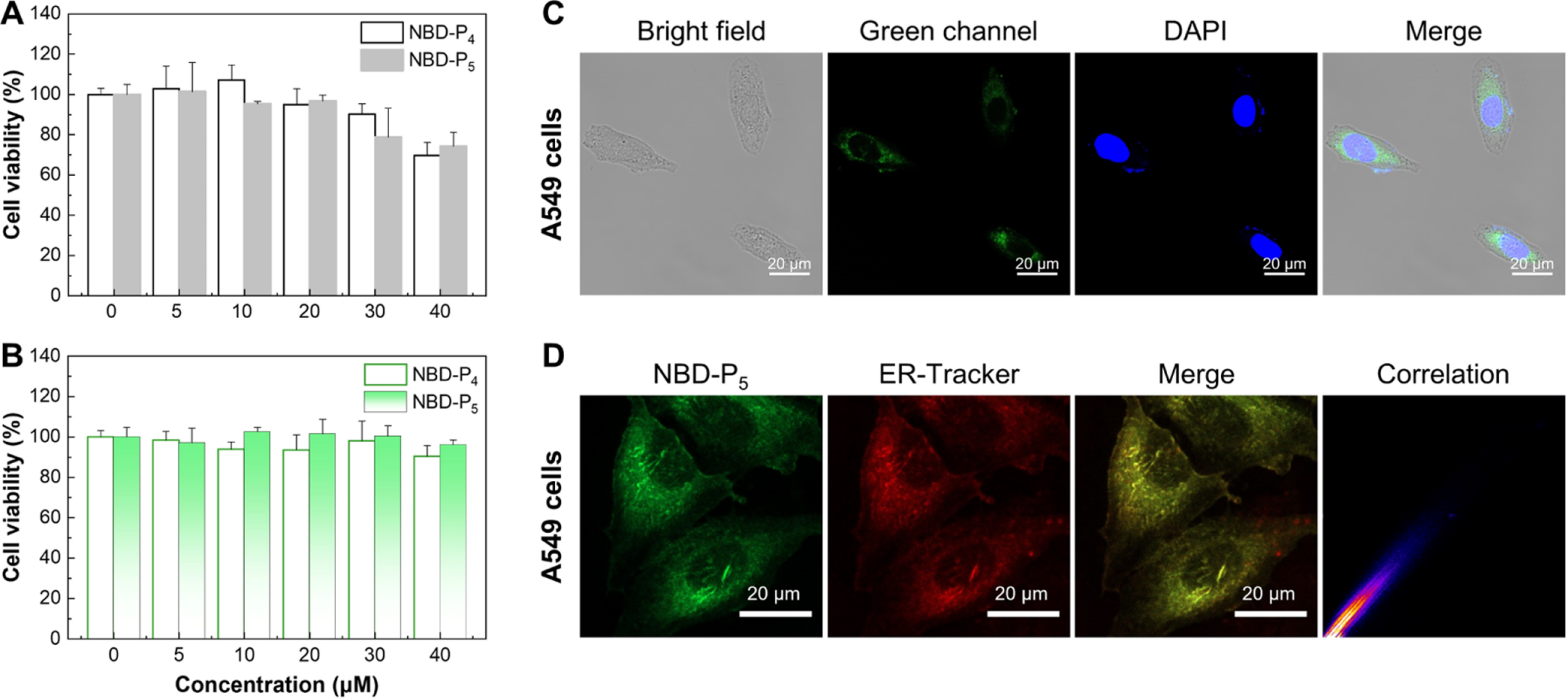
NBD-P_5_ is non- or weakly cytotoxic and can enter the cells to image HO-1. Cytotoxic effect of NBD-P_n_ in (A) normal cell line HK-2 cells and (B) cancer cell line A549 cells. (C) Confocal microscopic images of NBD-P_5_ in A549 cells. The cells were treated with NBD-P_5_ (10 μM) for 2 h, fixed, and costained with the nuclear staining dye DAPI for 20 min. (D) Colocalization of NBD-P_5_ and ER-Tracker red in A549 cells. Cells were incubated with NBD-P_5_ (10 μM) for 2 h and then costained with ER-Tracker red (400 nM) for 20 min. Pearson’s correlation coefficient was evaluated by ImageJ.

Before proceeding to the cellular imaging of HO-1, the cytotoxic effect of NBD-P*_n_* in cancerous A549 and normal HK-2 cell lines was evaluated. As demonstrated in Figure 4A,B, both probes showed negligible cytotoxicity. For example, treatment with up to 40 μM NBD-P*_n_* did not result in a significant decrease in cell viability, with as much as 70% viability being retained. Given its higher fluorescence responsiveness toward HO-1 in solution, NBD-P_5_ is represented as a better candidate probe and was selected to treat human lung adenocarcinoma A549 cells, as this cell type was reported to constitutively express HO-1.^33^ Flow cytometry analysis of the uptake of NBD-P_5_ by A549 cells was time-dependent and increased rapidly in the first 1 h, followed by a slow increase over the time course of the 2.5 h study (Figure S17). Upon incubation with NBD-P_5_, green fluorescence from the probe was mainly distributed around the nucleus, suggesting that NBD-P_5_ can enter the cells to image HO-1 (Figure 4C). Considering that HO-1 is mainly located at the endoplasmic reticulum, we then performed a colocalization experiment to check the endoplasmic reticulum targeting ability of NBD-P_5_. To our delight, there is an excellent overlap between the green fluorescence of NBD-P_5_ and the red fluorescence of ER-Tracker, with a high Pearson’s correlation coefficient of 0.97 (Figure 4D). In comparison, colocalization of NBD-P_5_ with either Lyso-Tracker or Mito-Tracker was not as significant as that with ER-Tracker (Figures S18 and S19). The results demonstrated that NBD-P_5_ was mainly located in the endoplasmic reticulum.

The successful imaging of HO-1 encouraged us to further explore whether NBD-P_5_ can respond to changes in HO-1 levels in live cells. The previous study by Fang and co-workers used tBHQ to induce HO-1 expression, and the results showed that HO-1 was successfully upregulated by about 1.6-fold.^14^ After checking the cytotoxic assay of tBHQ from the literature,^34^ we decided to use hemin^35^ and zinc(II) protoporphyrin (ZnPP)^36,37^ to upregulate and downregulate the intracellular level of HO-1, respectively. Before treating cells with hemin or ZnPP, their cytotoxic was measured (Figure S20). According to the cytotoxic result, the dosage was determined to be 50 and 1 μM for hemin and ZnPP, respectively.

**Figure 5:**
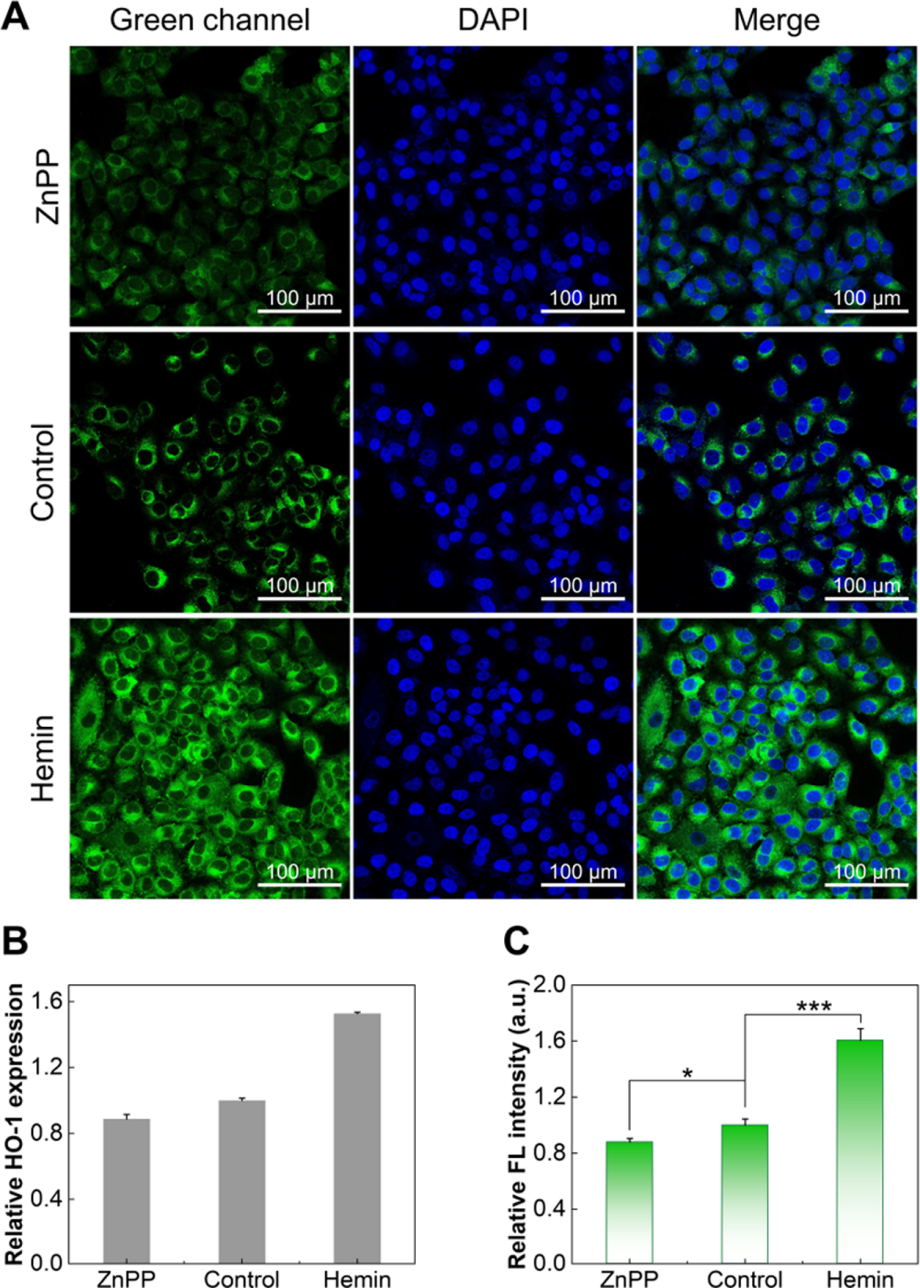
Assay of HO-1 levels by NBD-P_5_. Measurement of HO-1 levels in live A549 cells by (A) NBD-P_5_ and (B) a HO-1 ELISA kit. A549 cells were pretreated with ZnPP (1 μM) or 0.2% DMSO for control or hemin (50 μM) for 24 h. The cells were then lysed and analyzed by the HO-1 ELISA kit or stained with NBD-P_5_ for 2 h (10 μM, cell nuclei were counterstained by DAPI for 20 min). (C) Relative fluorescence intensity in cells in figure a, quantified by ImageJ (means ± SD, * *P* < 0.05, *** *P* < 0.001).

By treatment of A549 cells with hemin, the expression of HO-1 was upregulated by around 1.53-fold, as determined by the commercially available HO-1 ELISA kit. In comparison, ZnPP treatment showed a 0.89-fold decrease in HO-1 expression (Figures 5B and S21). Note that complex sample preparation steps and a long incubation time (around 3 h) are required for using the ELISA kit. We then tested the ability of NBD-P_5_ to report HO-1 concentrations in live A549 cells. As shown in Figure 5A, a significantly stronger green fluorescence was observed for the group treated with hemin, and the observation is comparable to the first fluorescent probe RBH reported in 2023 that works in live cells.^14^ Those incubated with ZnPP showed a relatively weaker fluorescence signal, with a comparison to fluorescence in the control cells. The observation demonstrates that NBD-P_5_ is suitable for reporting on levels of HO-1 in live cells. The quantitative analysis of average fluorescence intensity in Figure 5A is summarized in Figure 5C, which showed good consistency with the result from the HO-1 ELISA kit. NBD-P_5_ is, therefore, a fluorescent probe that can report live-cell HO-1 levels in a catalysis-independent manner; it quickly and highly responds to HO-1, eliminating the need for large sample amounts, long incubation times, and reagent additions, including biliverdin reductase and NADPH.

## Conclusions

In summary, we demonstrated that fluorescent probes based on a peptide derived from the natural HO-1-binding protein caveolin-1 provided a simple but effective approach for HO-1 detection. The prototype NBD-P_5_ could be used to report changes in HO-1 levels in live cells and had the advantage that it could be readily modified by simply replacing the fluorophore. Compared to the two previous HO-1 fluorescent probes, NBD-P_5_ acts independently of the catalytic activity of HO-1, which often requires the consumption of a large amount of the substrate along with a long incubation time. Therefore, it enables the fast and sensitive detection of HO-1. Our design also avoids the consumption of NADPH, O_2_, and biliverdin reductase during the detection; however, strategies based on the catalytic activity of HO-1 require their presence for HO-1 to catalyze. It is known that the O_2_ level is low in TME, and HO-1 is commonly expressed at a high level in tumors. Therefore, the development of probes for HO-1 in an enzyme-activity-independent manner is beneficial, which can be translated into powerful tools for further dissecting HO-1 functions.

## Supporting Information Available

General information; detailed experimental and synthetic methods; compound characterization; absorbance and fluorescence spectrum; cell culture; cell fluorescence imaging; flow cytometric analysis; and enzyme-linked immunosorbent assay (PDF)

## Abbreviations

HO-1: heme oxygenase-1
heme: Fe(III) protoporphyrin IX
HPLC: high-performance liquid chromatography
FRET: fluorescence resonance energy transfer
NBD: nitrobenzoxadiazole
MM/GBSA: molecular mechanics, general Born surface area
HSA: human serum albumin
BSA: bovine serum albumin
ZnPP: zinc(II) protoporphyrin
