# Supplementary material for: NADPH-Independent Fluorescent Probe for Live-Cell Imaging of Heme Oxygenase-1
Source: ACS Sens. 2025 Jan 2;10(1):499–506. doi: 10.1021/acssensors.4c02978 (PMC11773557; doi:10.1021/acssensors.4c02978)
Supplement: Supplementary file 1 — se4c02978_si_001.pdf [file se4c02978_si_001.pdf]

Supporting Information for:

An NADPH-independent fluorescent probe for live-cell imaging of heme oxygenase-1

Liang Li[1], Xuanyi Lu[1], Qiyuan He[1], Chao Shu[2], Edward R. H. Walter[3]*, Lin Wang[4]*, Nicholas J. Long[3]*, and Lijun Jiang[1]*

1. Hubei Key Laboratory of Genetic Regulation & Integrative Biology, Key Laboratory of Pesticide and Chemical Biology of Ministry of Education, School of Life Sciences, Central China Normal University, Wuhan 430079, China.
2. State Key Laboratory of Green Pesticide, College of Chemistry, Central China Normal University, Wuhan 430079, China.
3. Department of Chemistry, Imperial College London, MSRH Building, White City Campus, London W12 0BZ, UK.
4. Institute of Systems Medicine, Chinese Academy of Medical Sciences, Suzhou 215028, China.

* Corresponding authors: **Lijun Jiang**, Hubei Key Laboratory of Genetic Regulation & Integrative Biology, Key Laboratory of Pesticide and Chemical Biology of Ministry of Education, School of Life Sciences, Central China Normal University, Wuhan 430079, China. Email: lijunjiang@ccnu.edu.cn. **Nicholas J. Long** (n.long@imperial.ac.uk) or **Edward R. H. Walter** (e.walter@imperial.ac.uk), Department of Chemistry, Imperial College London, MSRH Building, White City Campus, London, UK. **Lin Wang**, Institute of Systems Medicine, Chinese Academy of Medical Sciences, Suzhou 215028, China. Email: wl@ism.cams.cn.

1. General considerations

Unless otherwise noted, all reagents and solvents were obtained from commercial suppliers and used without further purification. The ultrapure water used was from Milli-Q® Direct 8. The on-resin peptides, P_4 and P_5, were ordered from GL Biochem Ltd (Shanghai, China). Human full-length recombinant heme oxygenase-1 were ordered from Proteintech (Wuhan, China). The cell line HK-2 and A549 were purchased from the Shanghai Cell Bank of Chinese Academy of Sciences (Shanghai, China). ER-Tracker red, Lyso-Tracker Red, and Mito-Tracker red were ordered from Beyotime Biotechnology (Shanghai, China). The human HO-1 ELISA kit (JL10932) was ordered from Jonln (Shanghai, China).

Thin-layer chromatography (TLC) was performed using precoated silica gel 60 F_{254} aluminium sheets. Column chromatography was conducted using silica gel and laboratory grade solvents manually. HPLC was carried out on Agilent 1260 Infinity II LC system. ^{1}H NMR and ^{13}C NMR were recorded on Qone Quantum-I-Plus 400 MHz and Varian Mercury plus 400 MHz NMR spectrometer, respectively. High-resolution mass spectrum was carried out on Agilent 6224 Accurate-Mass TOF LC/MS. Absorption spectra were measured on SPECORD 210 PLUS UV/VIS spectrophotometer (Analytic Jena, Germany). Fluorescence spectra were measured on FLS1000 steady-state and transient fluorescence spectrometer (Edinburgh, UK). Cytotoxicity assay was measured by a BioTek Synergy-2 fully automated microplate reader. Flow cytometric analyses were obtained from the BD FACSVerse. (Becton, Dickinson and Company, USA). Cell imaging experiments were measured by ZEISS LSM 980 with Airyscan 2 confocal laser scanning microscope.

2. Synthesis and characterization

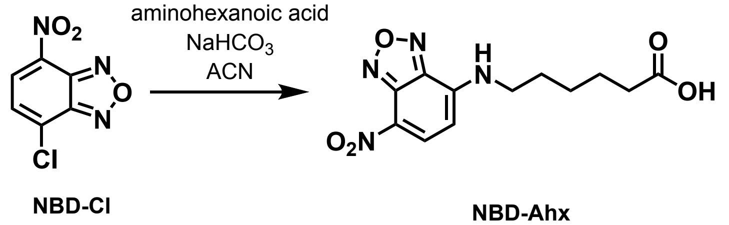

NBD-Ahx was synthesized following a modified literature procedure.[1] A solution of 4-chloro-7-nitrobenzofurazan (500 mg, 2.51 mmol) in ACN (6 mL) was added to the solution of aminohexanoic

acid (395 mg, 3.01 mmol) and $NaHCO_3$ (633 mg, 7.53 mmol) in deionized water (6 mL) at 65 °C. The resulting solution was stirred at 65 °C. Progress of the reaction was monitored by TLC. When the reaction was complete, the mixture was allowed to cool to room temperature. Organic solvents in the reaction mixture were evaporated under reduced pressure. pH of the remaining solution was adjusted to 2 using 1.0 M HCl, which was then extracted with EA for three times. The organic phase was combined and dried using anhydrous $MgSO_4$, and then filtered. The filtrate was evaporated to dryness, and the residue was chromatographed on silica gel using MeOH-DCM mixture as the eluent to give NBD-Ahx as a yellow solid (Yield: 43.4%).

^{1}H NMR (400 MHz, DMSO-d_6) δ: 11.96 (s, 1H), 9.57 (s, 1H), 8.51 (d, J = 8.9 Hz, 1H), 6.41 (d, J = 9.0 Hz, 1H), 3.45 (t, J = 7.1 Hz, 2H), 2.21 (t, J = 7.3 Hz, 2H), 1.68 (m, J = 7.4 Hz, 2H), 1.54 (m, J = 7.3 Hz, 2H), 1.37 (m, J = 8.5 Hz, 2H).

^{13}C NMR (101 MHz, DMSO-d_6) δ: 175.03, 145.74, 144.97, 144.68, 138.43, 120.89, 99.65, 43.78, 34.14, 27.89, 26.47, 24.69.

HRMS (*m/z*) calcd. for $C_{12}H_{14}N_4O_5$ $[M+H]^+$ 295.1037, found 295.1037; calcd. for $[M+Na]^+$ 317.0856, found 317.0857; calcd. for $[2M+Na]^+$ 611.1821, found 611.1822.

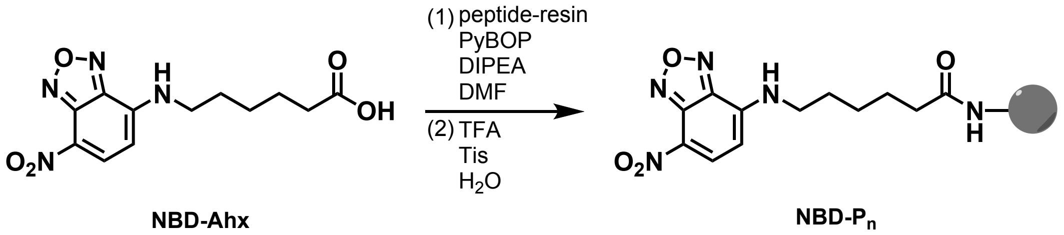

NBD-P$_n$ was synthesized similarly according to our previous report.[2] DIPEA (44 mg, 0.34 mmol) was added to the solution of NBD-Ahx (50 mg, 0.17 mmol) and (benzotriazol-1-yloxy)tripyrrolidinophosphonium hexafluorophosphate (PyBOP, 88 mg, 0.17 mmol) in anhydrous DMF (3 mL). After stirring for 5 min, the solution was added to the appropriate peptide (P_4/P_5, 0.085 mmol) on a 12 mL solid-phase extraction cartridge with PE frits. The peptide was pre-swelled in DMF overnight, and the chamber was drained before addition of the solution. The resulting mixture was agitated on a shaker for 2 h, and the solution was then filtered and washed with DMF for three times. A second round of coupling was performed according to the same procedures. Afterwards, the solution was filtered and washed as described above.

Resin cleavage. A mixture of TFA/TIPS/H_2O 95:2.5:2.5 (TFA: trifluoroacetic acid; TIPS: triisopropylsilane) was added to the crude resin shrunken with diethyl ether. The mixture was agitated on a shaker for 2.5 h. The resin was then washed with MeOH for three times and removed by filtration. The combined filtrate was concentrated to 1 mL gently under reduced pressure. The NBD-P_n crude was precipitated by dilution with 40 mL ice-cold diethyl ether and collected by centrifugation. The yellow precipitates were washed twice more with ether and then re-dissolved and purified by semi-preparative HPLC.

HPLC. HPLC was performed on Agilent 1260 Infinity II LC system using either an Agilent Zorbax SB-C18 or a Waters Symmetry C18 column. Deionized water containing 0.1% TFA was used as the eluent A and MeOH as the eluent B. 280 and 254 nm were selected to record the chromatograms. Identity of the target peak was confirmed with Agilent 6224 Accurate-Mass TOF LC/MS.

NBD-P_4. Retention time, 30.903 min. HRMS (m/z): calcd. for $C_{50}H_{52}N_{10}O_{10}$ $[M+Na]^+$ 975.3760, found 975.3768.

NBD-P_5. Retention time, 21.513 min. HRMS (m/z): calcd. for $C_{56}H_{64}N_{14}O_{11}$ $[M+H]^+$ 1109.4952, found 1109.4952.

3. Computational modeling

The complex structure of HO-1 with the pentapeptide P_5 was first generated using the online server AlphaFold3.[3] The complex structure of HO-1 with P_4 was then constructed by deleting the terminal arginine. Protein structure preparation and optimization were performed using the Protein Preparation Wizard [4] program in Schrödinger 2023-3 (Schrödinger Inc.). Subsequently, the C-terminal carboxyl group of the peptides was modified to amide using the 3D structure editor in Maestro, followed by 6 rounds of energy minimization iterations using the Prime module in Schrödinger 2023-3 to further optimize the structures. Based on the complex structures of the peptide ligands with HO-1, we employed the Glide [5] module in Schrödinger 2023-3 to perform constrained docking to dock the fluorescent probes to HO-1. In the docking process, the length of the inner box was set to 12 Å, and the docking precision was set to standard precision. We carried out 4-fold enhanced sampling and generated 100,000 initial poses in the initial phase, and only the best-scoring docking pose was retained. Finally, we utilized the Molecular Mechanics, General Born Surface Area (MM/GBSA) method in the

Prime module to optimize the residues within 5 Å around the binding interface of the complex and calculate the binding free energy. The predicted complex structures were visualized and analyzed by PyMol.[6]

4. Photophysical measurements

All spectra were measured using a stock solution of the probes (1 mM in DMSO) which was diluted with pH 7.4 PBS buffer to the desired concentration. Luminescence titration was performed by measuring emission of NBD-P_n in pH 7.4 PBS buffer with the gradually-increased concentration of HO-1. pH sensitivity (or stability) was performed by measuring emission of NBD-P_n in aqueous solution with varying pH conditions. Aqueous solution with different pH values was prepared by adding 1 M HCl or NaOH to deionized water. Selectivity assay was performed by measuring emission of NBD-P_n in pH 7.4 PBS buffer in presence of varying analytes including HO-1, common proteins, amino acids, and biologically relevant ions. All measurements were conducted using a 700 µL quartz cuvette and within a minute after addition of the analyte.

5. Cell culture

HK-2 human normal kidney cells and A549 lung cancer cells were used in this study. HK-2 cells were cultured in DMEM-F12 supplemented with 10% fetal bovine serum, 1% penicillin, and 1% streptomycin in a CO_2 incubator at 37°C. A549 cells were cultured in high-glucose DMEM supplemented with 10% fetal bovine serum, 1% penicillin, and 1% streptomycin in a CO_2 incubator at 37°C.

6. Cytotoxicity assay

Cytotoxicity of NBD-P_4, NBD-P_5, hemin and ZnPP was evaluated by MTT assay. Briefly, 5×10^3 cells/100 µL per well were seeded in 96-well plates and incubated for 24 h in a CO_2 incubator at 37°C. Different concentrations of the analyte were added to the cells. After overnight incubation, 10 µL of MTT solution (5 mg/mL) was added to each well, and the plates were incubated for another 4 h. Then, 100 µL of DMSO was added to dissolve the formed formazan crystal. After shaking for 30 min,

absorbance was read at 570 nm using a BioTek Synergy-2 fully automated microplate reader. Cell viability was calculated according to the following equation:

$$\text{Viability (\%)} = (OD_i/OD_c) \times 100\%$$

Where OD_i and OD_c refers to optical density of the surviving cells treated with or without the analyte, respectively.

7. Enzyme linked immunosorbent assay (ELISA)

A549 cells (8×10^3 cells per well) were seeded in 6-well plates and incubated in a CO_2 incubator at 37°C for 24 h. Cells were treated with 50 μM hemin, 1 μM ZnPP or DMSO. After 24 h incubation, cells were washed with PBS, lysed with NP-40 buffer containing 1.0 mM PMSF, followed by centrifugation (12000 rpm, 5 min) to harvest the supernatant. The assay was carried out by strictly following instructions provided. Absorbance at 450 nm was recorded using the BioTek Synergy-2 Microplate Reader to calculate the HO-1 concentration under different treatments.

8. Flow cytometry analysis

A549 cells were first seeded into 12-well plates (1.0×10^5 cells/well) and incubated overnight to achieve adherence. Then, 10 μM NBD-P_5 was added, and incubated for different times. The cells were washed twice with cold PBS and harvested by trypsinization, followed by centrifugation (1500 x g, 5.0 min). The obtained cells were re-suspended in PBS and analyzed using flow cytometry (BD FACSVerse).

9. Fluorescence imaging

A549 cells (8×10^3 cells per well) were seeded into confocal dish and incubated in a CO_2 incubator at 37°C for 24 h. Cells were treated with 50 μM hemin, 1 μM ZnPP or DMSO. After 24 h incubation, 10 μM NBD-P_5 was added and incubated with the cells for 2 h. The cells were then fixed with 4% polyoxymethylene for 20 min. Afterwards, the nuclei were stained using DAPI for 20 min. Fluorescence images were measured by ZEISS LSM 980 with Airyscan 2 confocal laser scanning microscope.

10. Colocalization experiment

A549 cells (8×10^3 cells per well) were seeded into confocal dish and incubated in a CO_2 incubator at 37°C for 24 h. After incubation with NBD-P_5 (10 μM) for 2 h, cells were co-stained with ER-Tracker red (400 nM) for 20 min, Lyso-Tracker red (60 nM) for 30 min, or Mito-Tracker red (50 nM) for 30 min. Fluorescence images were measured by ZEISS LSM 980 with Airyscan 2 confocal laser scanning microscope.

Supplementary Figures

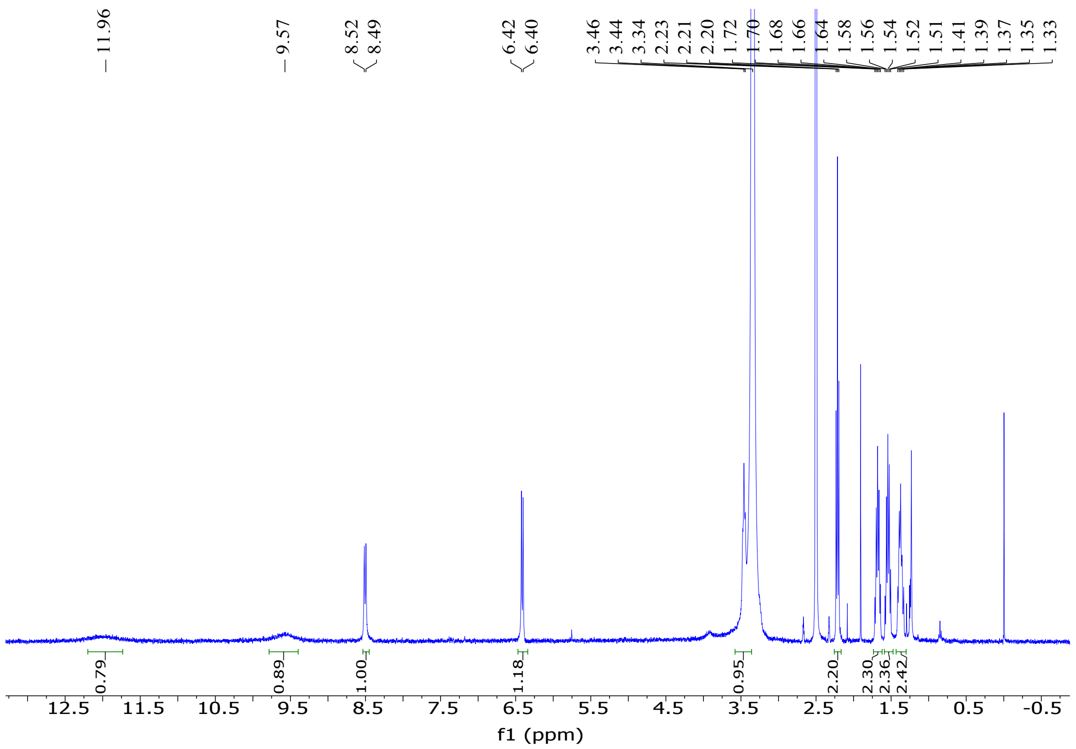

Figure S1. ^{1}H NMR spectrum of NBD-Ahx (400 MHz, d_6-DMSO).

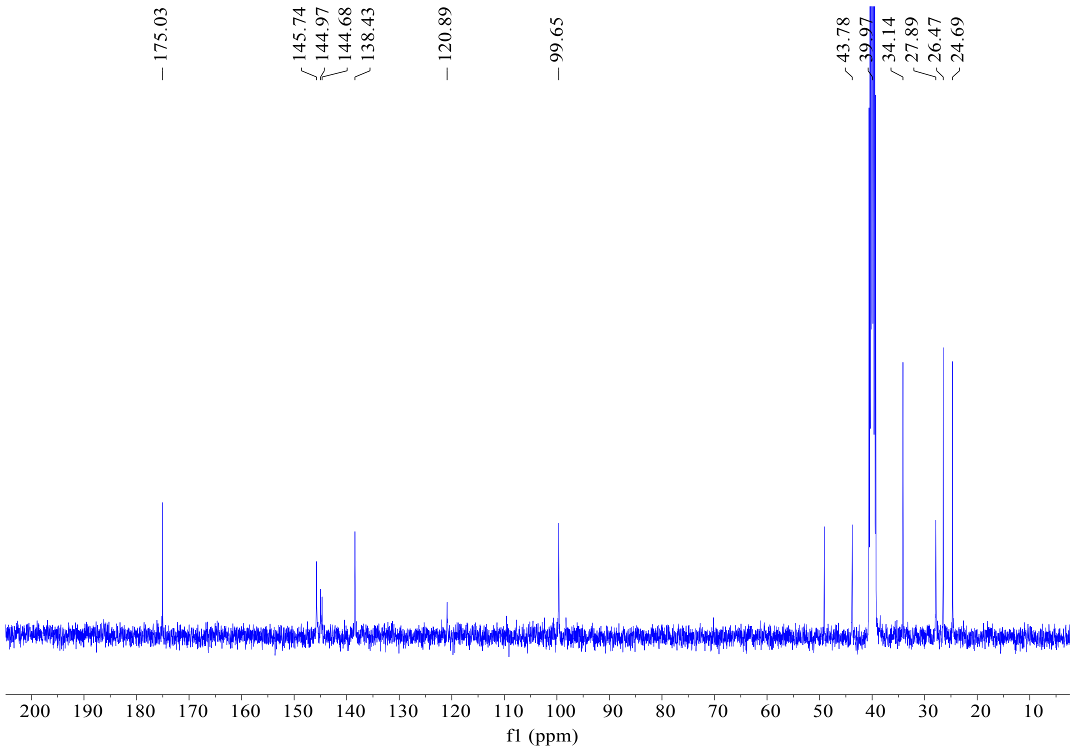

Figure S2. ^{13}C NMR spectrum of NBD-Ahx (101 MHz, d_6-DMSO).

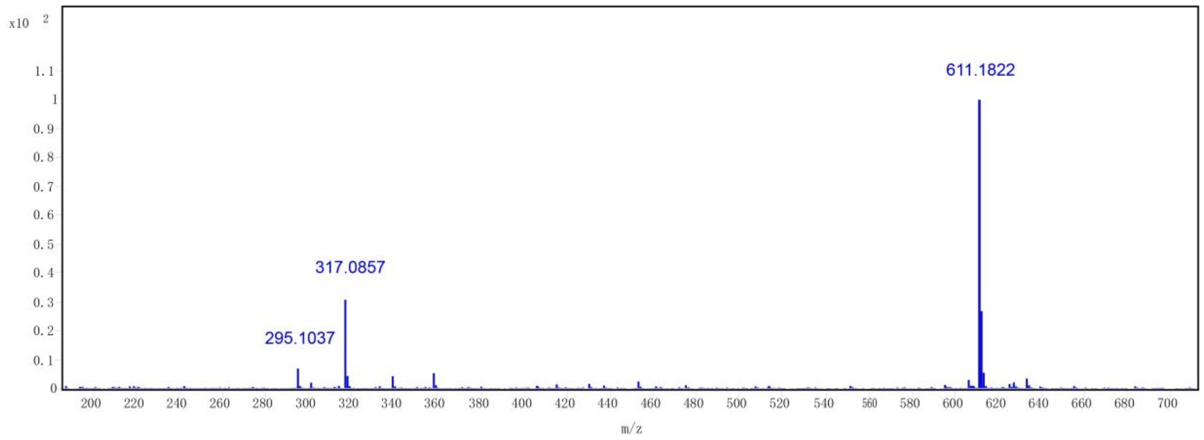

Figure S3. High-resolution mass spectrum of NBD-Ahx.

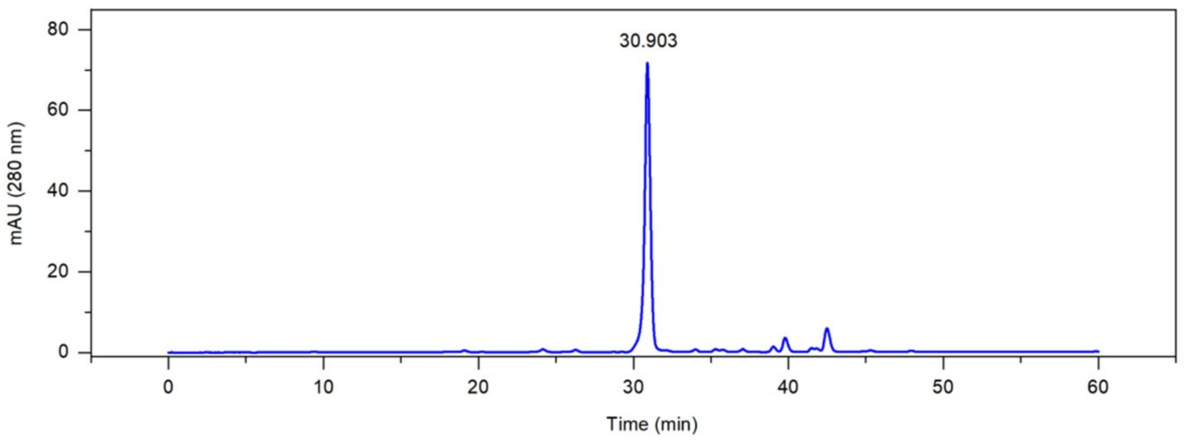

Figure S4. HPLC chromatogram of $\mathbf{NBD\text{-}P_4}$ recorded at 280 nm.

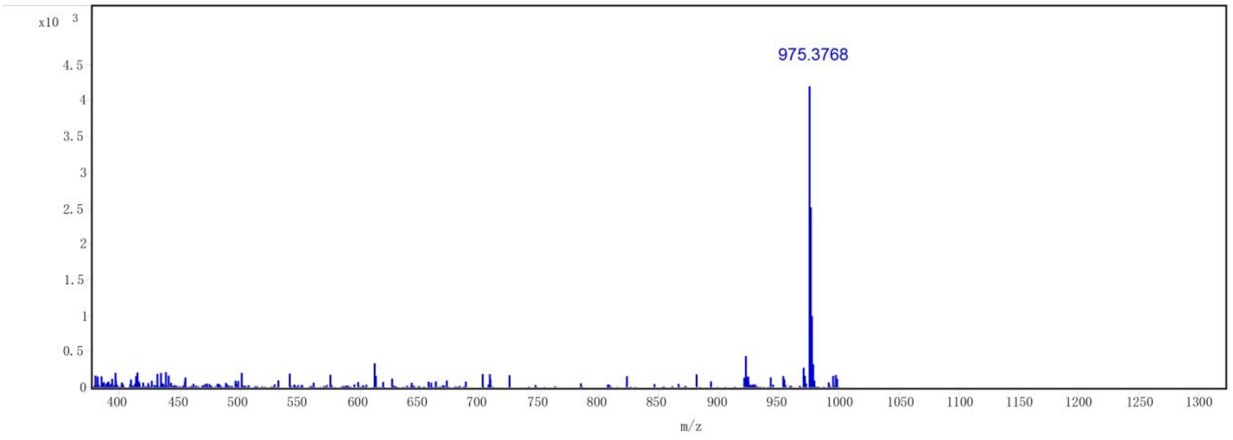

Figure S5. High-resolution mass spectrum of $\mathbf{NBD\text{-}P_4}$.

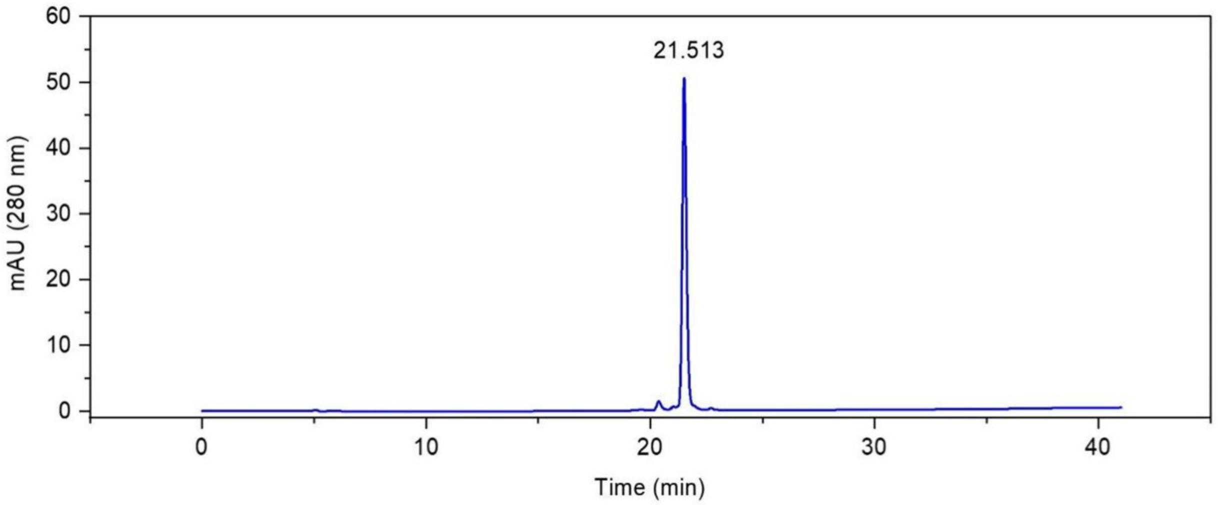

Figure S6. HPLC chromatogram of **NBD-P$_5$** recorded at 280 nm.

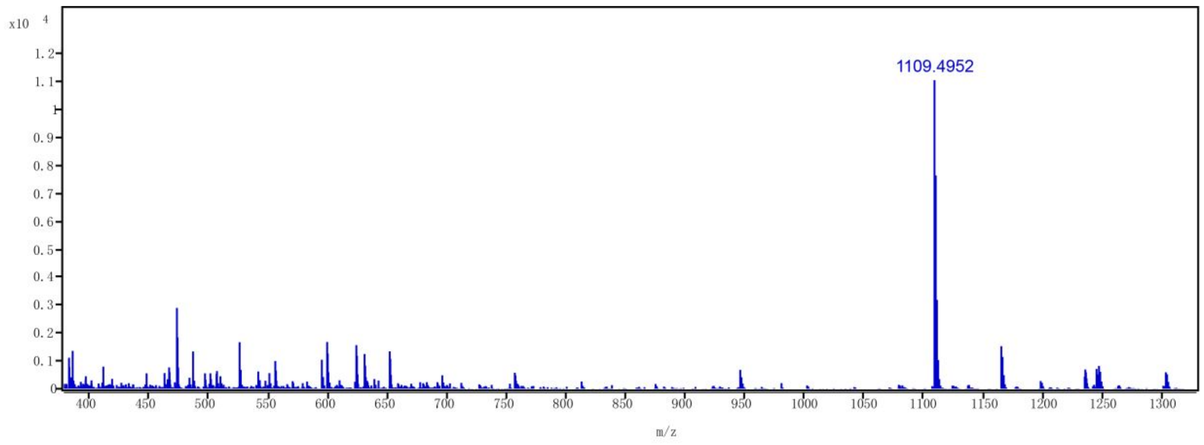

Figure S7. High-resolution mass spectrum of **NBD-P$_5$**.

A

10	20	30	40	50
MERPQPDSMP	QDLSEALKEA	TKEVHTQAEN	AEFMRNFQKG	QVTRDGFKLV
60	70	80	90	100
MASLYHIYVA	LEEEIERNKE	SPVFAPVYFP	EELHRKAALE	QDLAFWYGPR
110	120	130	140	150
WQEVIPYTPA	MQRYVKRLHE	VGRTEPELLV	AHAYTRYLGD	LSGGQVLKKI
160	170	180	190	200
AQKALDLPSS	GEGLAFFTFP	NIASATKFKQ	LYRSRMNSLE	MTPAVRQRVI
210	220	230		
EEAKTAFLLN	IQLFEELQEL	LTHDTKDQSP	SRA	

B

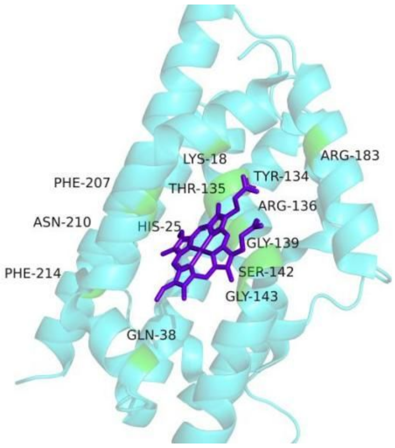

Figure S8. Structural features of HO-1. (A) Amino acid sequence of the major structural motifs of HO-1. (B) X-ray crystal structure of human HO-1 in complex with heme (PDB ID: 1N45).[7] HO-1 was rendered in ribbon, and the major heme binding sites in HO-1 were highlighted and shown in green. These binding sites include LYS-18, HIS-25, GLN-38, TYR-134, THR-135, ARG-136, GLY-139, SER-142, GLY-143, ARG-183, PHE-207, ASN-210, PHE-214. Heme is shown in purple.

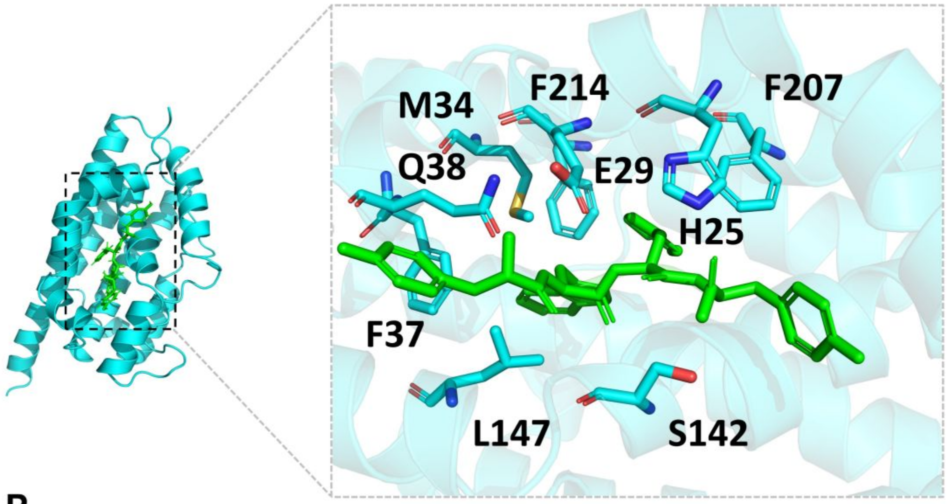

P_4
Binding Energy: -108.04 (kcal/mol)

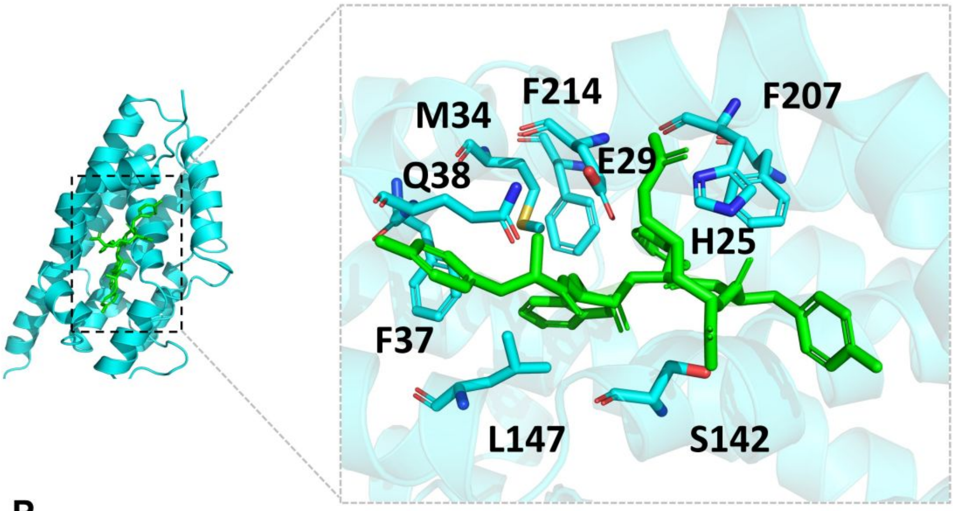

P_5
Binding Energy: -119.35 (kcal/mol)

Figure S9. The predicted binding poses and binding energies between HO-1 and P_n, obtained by computational modeling. P_n is shown in stick model in green. Key residues in HO-1 forming strong interactions with P_n are labeled and shown in stick.

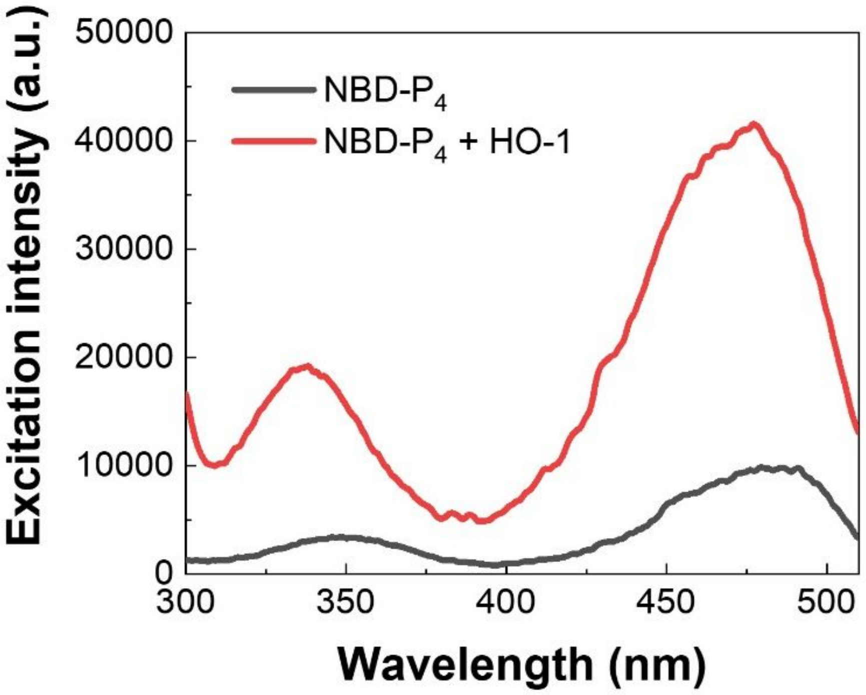

Figure S10. Excitation profile of NBD-P_4 recorded at λ_{em} = 535 nm before and after addition of HO-1. λ_{ex} = 475 nm, [NBD-P_4] = 2.0 μM, [HO-1] = 4.5 μM.

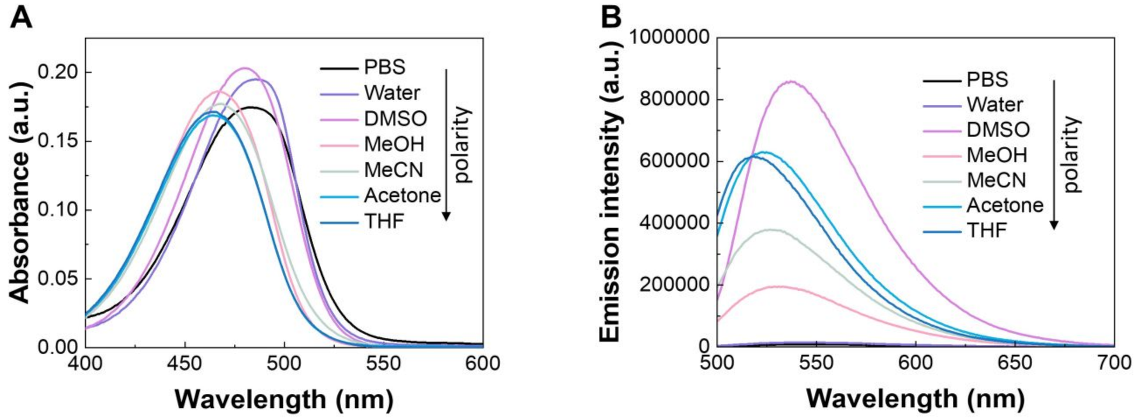

Figure S11. (A) Absorption and (B) emission spectra of NBD-P_5 at different polarity conditions. λ_{ex} = 475 nm, [NBD-P_5] = 2.0 μM.

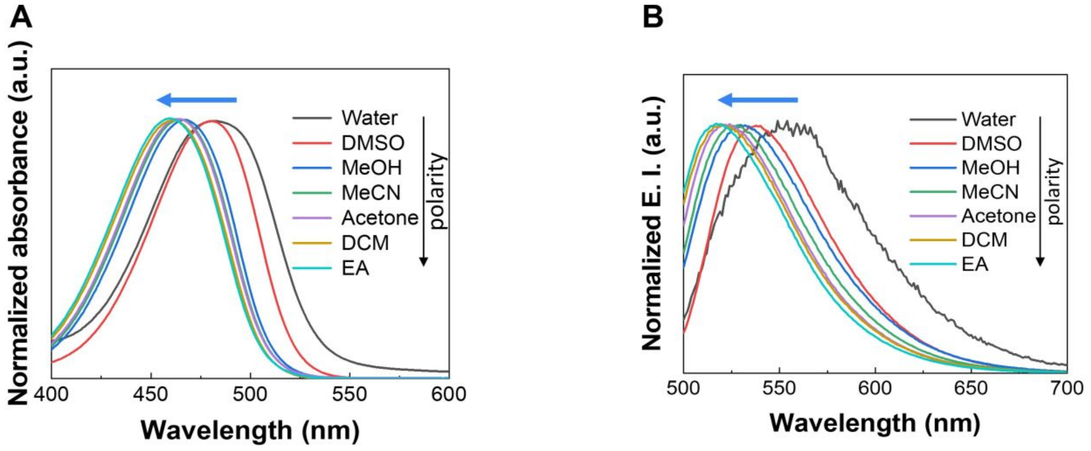

Figure S12. (A) Normalized absorption and (B) emission spectra of NBD-P_4 at different polarity conditions. λ_{ex} = 475 nm, [NBD-P_4] = 2.0 μM.

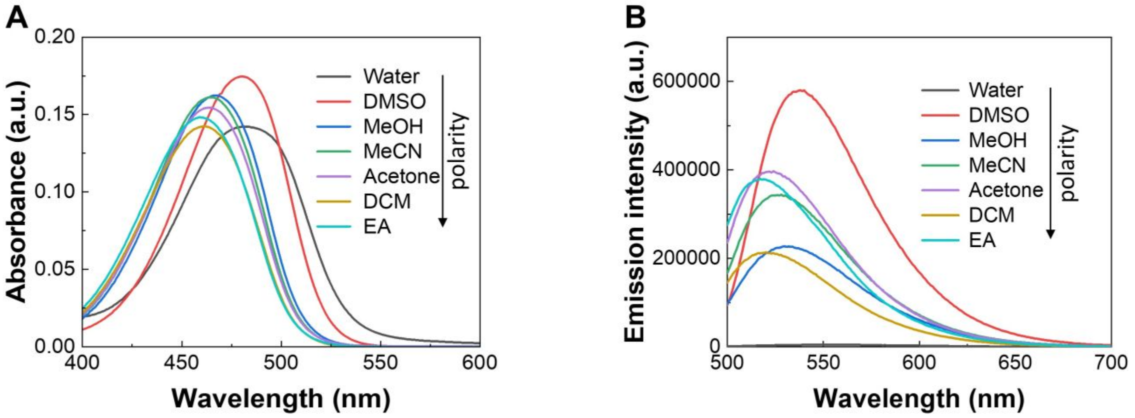

Figure S13. (A) Absorption and (B) emission spectra of NBD-P_4 at different polarity conditions. λ_{ex} = 475 nm, [NBD-P_4] = 2.0 μM.

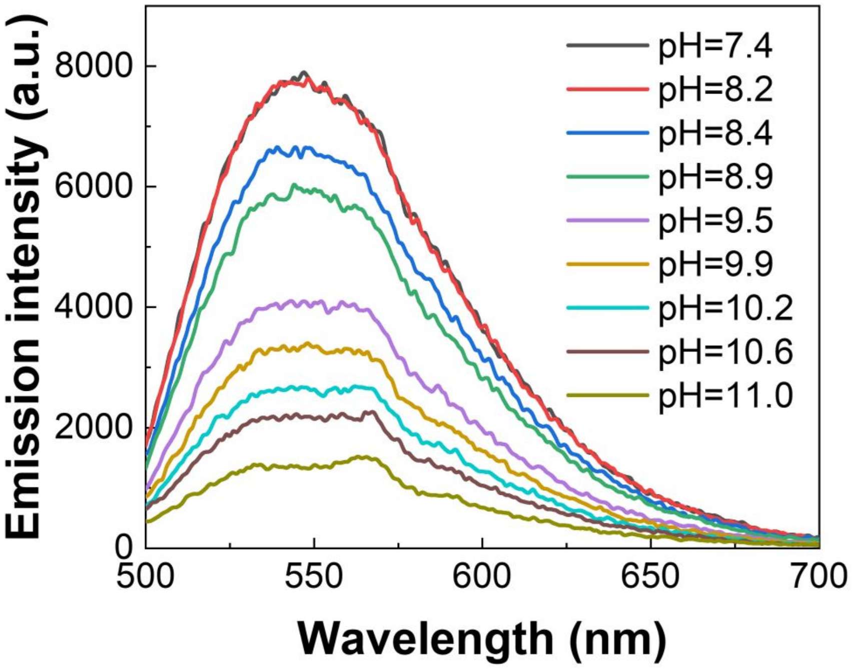

Figure S14. Emission profile of NBD-P_5 under basic pH conditions. A decline of emission intensity was shown when pH exceeds 8.2. λ_{ex} = 475nm, [NBD-P_5] = 2 μM.

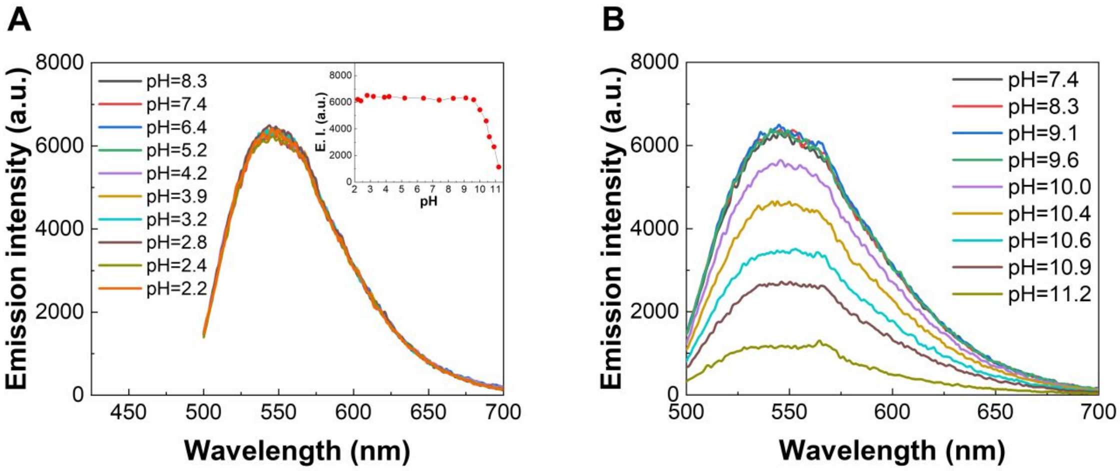

Figure S15. (A) Emission profile of NBD-P_4 at pH ranges from 8.3-2.2. Inlet: emission intensity of NBD-P_4 at 548 nm under different pH conditions. (B) A decline of emission intensity was shown when pH exceeds 9.6. λ_{ex} = 475nm, [NBD-P_4] = 2 μM.

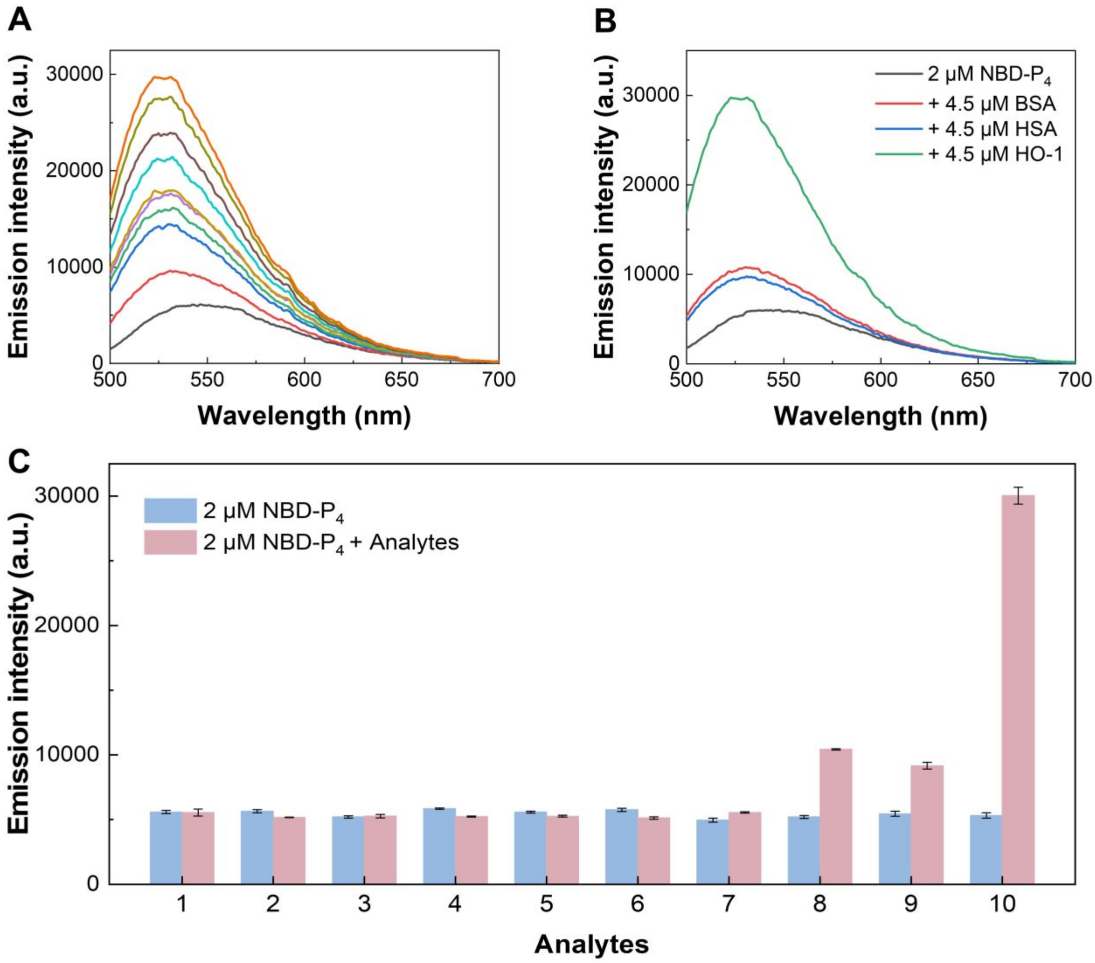

Figure S16. Response and selectivity of NBD-P_4 to HO-1. Emission profile of NBD-P_4 in responding to (A) recombinant HO-1 with increasing concentrations (0, 0.5, 1, 1.5, 2, 2.5, 3, 3.5, 4, 4.5 μM). (B) HO-1, BSA and HSA under the same concentration. (C) Fluorescence response of NBD-P_4 to HO-1 among biological relevant ions, amino acids, and common proteins. Bars represent fluorescence intensity of NBD-P_4 at 526 nm before and after addition of analytes. The concentration is 1 mM for 1. NaCl, 2. KCl, 3. $NaHCO_3$, 4. KH_2PO_4, 5. Ala, 6. Lys, 7. GSH. The concentration is 4.5 μM for 8. BSA, 9. HSA, 10. HO-1. [NBD-P_4] = 2 μM. λ_{ex}= 475 nm.

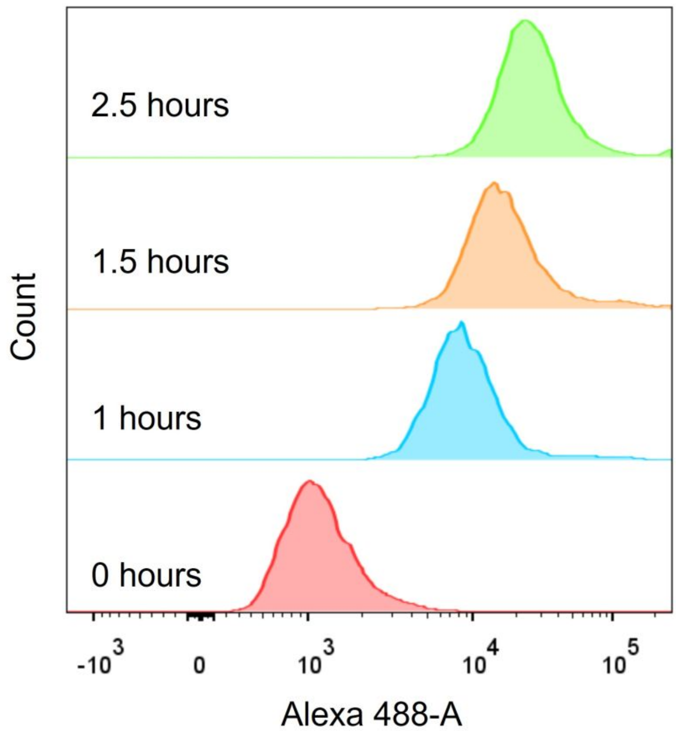

Figure S17. Time-dependent cellular uptake of NBD-P_5 by A549 cells via flow cytometric analysis. [NBD-P_5] = 10 μM.

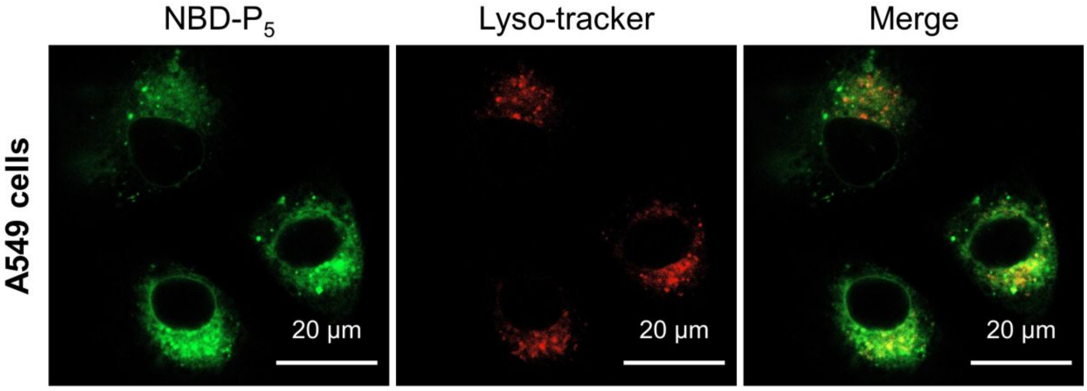

Figure S18. Colocalization of NBD-P_5 and Lyso-Tracker red in A549 cells. Cells were incubated with NBD-P_5 (10 μM) for 2 h and then co-stained with Lyso-Tracker red (60 nM) for 30 min.

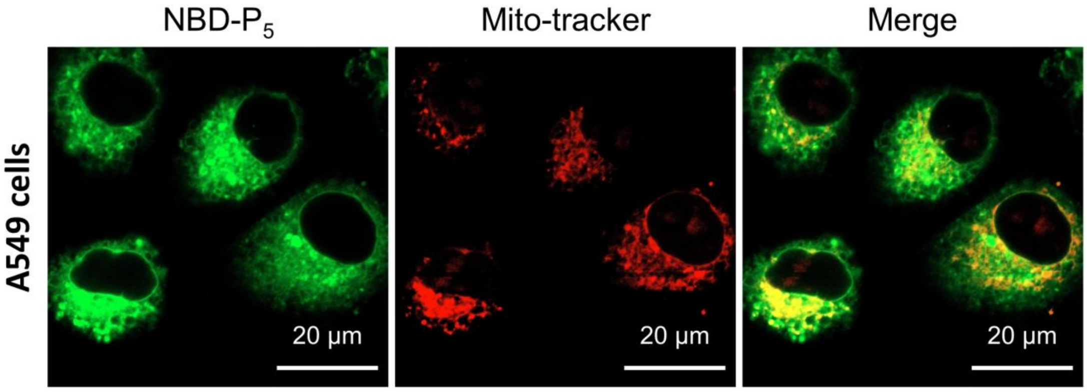

Figure S19. Colocalization of NBD-P_5 and Mito-Tracker red in A549 cells. Cells were incubated with NBD-P_5 (10 μM) for 2 h and then co-stained with Mito-Tracker red (50 nM) for 30 min.

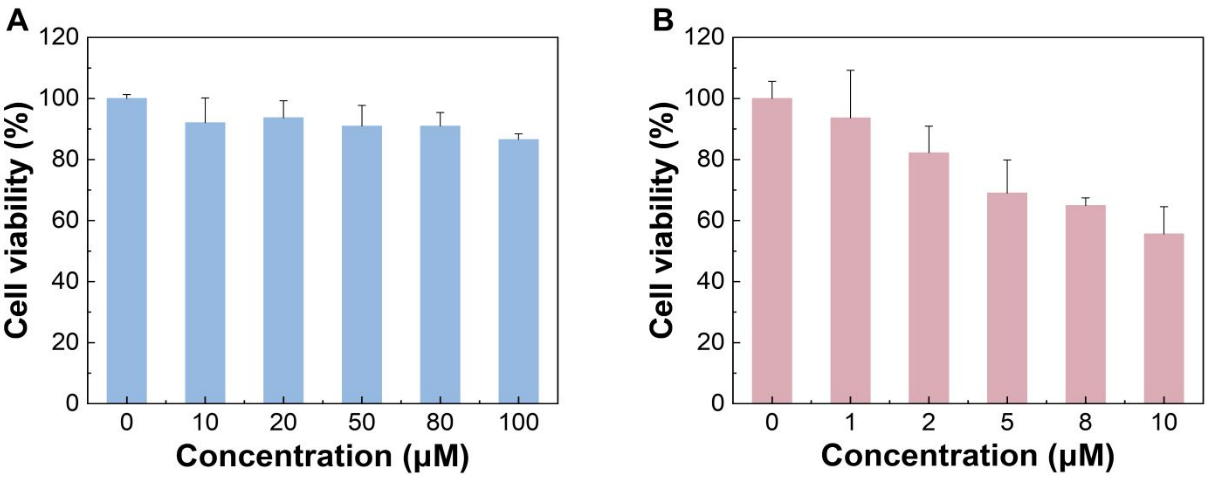

Figure S20. Cytotoxic effects of (A) hemin and (B) ZnPP on A549 cells.

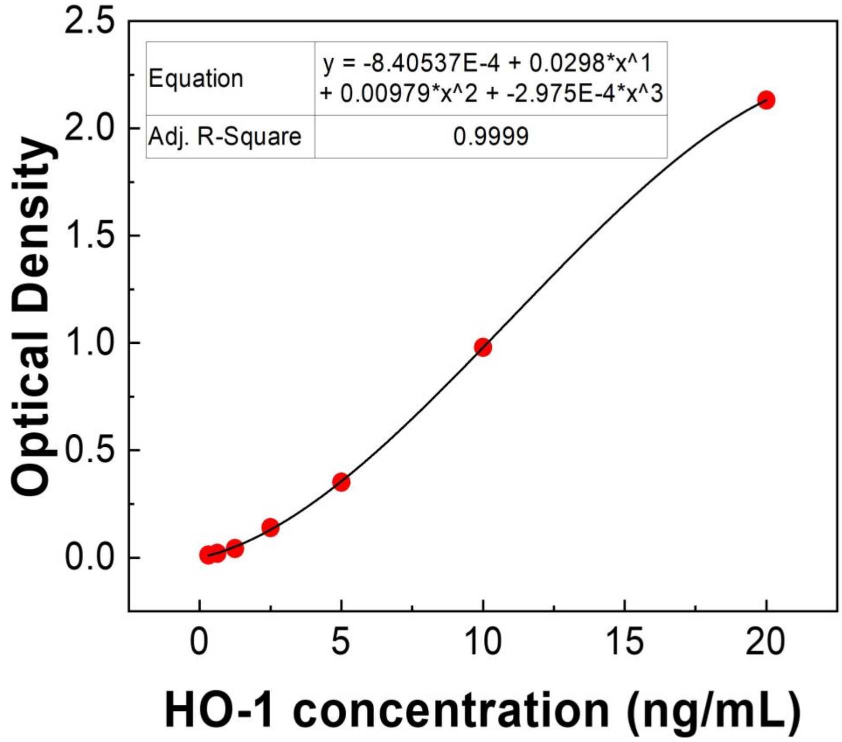

Figure S21. Standard curve of ELISA for the concentration measurement of HO-1.

References

(1) Woodland, J. G.; Hunter, R.; Smith, P. J.; Egan, T. J. Shining new light on ancient drugs: preparation and subcellular localisation of novel fluorescent analogues of Cinchona alkaloids in intraerythrocytic Plasmodium falciparum. *Org. Biomol. Chem.* **2017**, *15*, 589-597.

(2) Jiang, L.; Lan, R.; Huang, T.; Chan, C.-F.; Li, H.; Lear, S.; Zong, J.; Wong, W.-Y.; Muk-Lan Lee, M.; Dow Chan, B.; Chan, W.-L.; Lo, W.-S.; Mak, N.-K.; Li Lung, M.; Lok Lung, H.; Wah Tsao, S.; Taylor, G. S.; Bian, Z.-X.; Tai, W. C. S.; Law, G.-L.; Wong, W.-T.; Cobb, S. L.; Wong, K.-L. EBNA1-targeted probe for the imaging and growth inhibition of tumours associated with the Epstein–Barr virus. *Nat. Biomed. Eng.* **2017**, *1*, 0042.

(3) Abramson, J.; Adler, J.; Dunger, J.; Evans, R.; Green, T.; Pritzel, A.; Ronneberger, O.; Willmore, L.; Ballard, A. J.; Bambrick, J.; Bodenstein, S. W.; Evans, D. A.; Hung, C.-C.; O'Neill, M.; Reiman, D.; Tunyasuvunakool, K.; Wu, Z.; Žemgulytė, A.; Arvaniti, E.; Beattie, C.; Bertolli, O.; Bridgland, A.; Cherepanov, A.; Congreve, M.; Cowen-Rivers, A. I.; Cowie, A.; Figurnov, M.; Fuchs, F. B.; Gladman, H.; Jain, R.; Khan, Y. A.; Low, C. M. R.; Perlin, K.; Potapenko, A.; Savy, P.; Singh, S.; Stecula, A.; Thillaisundaram, A.; Tong, C.; Yakneen, S.; Zhong, E. D.; Zielinski, M.; Žídek, A.; Bapst, V.; Kohli, P.; Jaderberg, M.; Hassabis, D.; Jumper, J. M. Accurate structure prediction of biomolecular interactions with AlphaFold 3. *Nature.* **2024**, *630*, 493-500.

(4) Sastry, G. M.; Adzhigirey, M.; Day, T.; Annabhimoju, R.; Sherman, W. Protein and ligand preparation: parameters, protocols, and influence on virtual screening enrichments. *J. Comput.-Aided Mol. Des.* **2013**, *27*, 221-234.

(5) Friesner, R. A.; Banks, J. L.; Murphy, R. B.; Halgren, T. A.; Klicic, J. J.; Mainz, D. T.; Repasky, M. P.; Knoll, E. H.; Shelley, M.; Perry, J. K.; Shaw, D. E.; Francis, P.; Shenkin, P. S. Glide: A New Approach for Rapid, Accurate Docking and Scoring. 1. Method and Assessment of Docking Accuracy. *J. Med. Chem.* **2004**, *47*, 1739-1749.

(6) The PyMOL Molecular Graphics System, Version 2.5.7 Schrödinger, LLC.

(7) Lad, L.; Schuller, D. J.; Shimizu, H.; Friedman, J.; Li, H.; Ortiz de Montellano, P. R.; Poulos, T. L. Comparison of the heme-free and -bound crystal structures of human heme oxygenase-1. *J. biol. chem.* **2003**, *278*, 7834-7843.
